# High‐resolution environmental and host‐related factors impacting questing *Ixodes scapularis* at their northern range edge

**DOI:** 10.1002/ece3.10855

**Published:** 2024-02-21

**Authors:** Kirsten E. Crandall, Virginie Millien, Jeremy T. Kerr

**Affiliations:** ^1^ Department of Biology University of Ottawa Ottawa Ontario Canada; ^2^ Department of Biology McGill University Montréal Québec Canada; ^3^ Redpath Museum McGill University Montréal Québec Canada

**Keywords:** climate, host diversity and abundance, *Ixodes scapularis*, mammal, *Peromyscus leucopus*

## Abstract

The geographic range of tick populations has expanded in Canada due to climate warming and the associated poleward range shifts of their vertebrate hosts. Abiotic factors, such as temperature, precipitation, and snow, are known to directly affect tick abundance. Yet, biotic factors, such as the abundance and diversity of mammal hosts, may also alter tick abundance and consequent tick‐borne disease risk. Here, we incorporated host surveillance data with high‐resolution environmental data to evaluate the combined impact of abiotic and biotic factors on questing *Ixodes scapularis* abundance in Ontario and Quebec, Canada. High‐resolution abiotic factors were derived from remote sensing satellites and meteorological towers, while biotic factors related to mammal hosts were derived from active surveillance data that we collected in the field. Generalized additive models were used to determine the relative importance of abiotic and biotic factors on questing *I. scapularis* abundance. Combinations of abiotic and biotic factors were identified as important drivers of abundances of questing *I. scapularis*. Positive and negative linear relationships were found for questing *I. scapularis* abundance with monthly mean precipitation and accumulated snow, but no effect was found for the relative abundance of white‐footed mice. Positive relationships were also identified between questing *I. scapularis* abundance with monthly mean precipitation and mammal species richness. Therefore, future studies that assess *I. scapularis* should incorporate host surveillance data with high‐resolution environmental factors to determine the key drivers impacting the abundance and geographic spread of tick populations and tick‐borne pathogens.

## Graphical abstract

High‐resolution environmental and host active surveillance data were concurrently evaluated for their impact on questing *Ixodes scapularis* abundances in Central Canada. Combinations of abiotic and biotic factors were identified as important drivers of abundances of questing *I. scapularis*, which included monthly mean precipitation, accumulated snow, and mammal species richness.

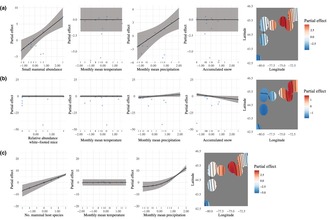

## 1 INTRODUCTION

Blacklegged ticks (*Ixodes scapularis*) are a disease vector of significant public health concern in the temperate regions of North America. Currently, this tick vector has a wide geographic range across Canada, with long‐established populations in Manitoba, Ontario, Quebec, New Brunswick, and Nova Scotia (Crandall et al., 2022; Guillot et al., 2020; Ogden et al., 2014; Wilson et al., 2022). In addition, *I. scapularis* is capable of transmitting various tick‐borne pathogens that cause tick‐borne diseases in humans, including anaplasmosis, babesiosis, and Lyme disease (Dibernardo et al., 2014; Nelder et al., 2021; Wilson et al., 2022). In response to changes in climate and land use, several environmental and host‐related factors may act as potential drivers in the establishment and poleward expansion of *I. scapularis* in Canada (Table S1; Alkishe et al., 2021; Bouchard et al., 2019; Ogden & Lindsay, 2016).

With climate warming, higher temperatures are expected to increase tick abundances through faster development rates and longer seasonal activity periods (Eisen et al., 2016; Ogden et al., 2004, 2021; Ogden & Lindsay, 2016). Laboratory studies on *I. scapularis* have found that extreme cold or hot temperatures have been associated with increased mortality rates and disturbed physiological processes, such as oviposition and egg mass development (Eisen et al., 2016; Fieler et al., 2021; Ogden et al., 2004). However, *I. scapularis* in nature may use microclimate refuges under leaf litter to avoid adverse weather conditions and maintain their optimal thermal thresholds (Linske et al., 2019; Volk et al., 2022). As a result, climate warming may affect *I. scapularis* populations in distinct ways depending on the temperature variability and extremes experienced locally at their range edges, with increased extirpation risk at the southern edge with very high temperatures and facilitated establishment at the northern range edge with warming temperatures (Ogden et al., 2013).

Abundant snow cover may also increase tick survival by providing an additional insulative layer from cold subzero temperatures. Snow cover alone or in combination with leaf litter have been found to increase overwintering survival by providing sufficient insulation to prevent inoculative freezing or desiccation (Linske et al., 2019; Volk et al., 2022). Summer nymphal *I. scapularis* densities have been found to increase after greater winter precipitation including snowfall (Hayes et al., 2015). In contrast, milder winters with intermittent snow cover may increase the risk of inoculative freezing in ticks, thereby limiting tick survival and densities the subsequent summer (Eisen et al., 2016; Linske et al., 2019; Volk et al., 2022).

Greater precipitation may maintain sufficient humidity within the microclimate leading to greater tick densities, while extended periods of low moisture may reduce tick survival and densities (Berger, Ginsberg, Dugas, et al., 2014; Berger, Ginsberg, Gonzalez, & Mather, 2014; Dumas et al., 2022). Hot, dry summer days have been found to increase mortality in *I. scapularis* due to greater water loss and desiccation stress (Burtis et al., 2016; Eisen et al., 2016). However, behavioral changes in ticks may mitigate their desiccation through modifications in their questing activity (Vail & Smith, 2002). Yet, increased levels of hydric stress may result in higher desiccation risk and lower *I. scapularis* abundances (Berger, Ginsberg, Gonzalez, & Mather, 2014; Burtis et al., 2016; Diuk‐Wasser et al., 2006).

Denser vegetation, especially in forested areas, may provide more suitable habitats for *I. scapularis* tick survival and development (Clow, Ogden, et al., 2017; Ginsberg et al., 2020; Mathisson et al., 2021; Schulze & Jordan, 2001). Several studies have found a positive association between *I. scapularis* and dense shrub vegetation (Mathisson et al., 2021). More specifically, the density of understory and shrubs were found to impact the risk of *I. scapularis* present in Ontario, with greater risk with low understory density and a medium to high relative abundance of shrubs due to its suitability for ticks and small mammals (Clow, Ogden, et al., 2017). In contrast, lower *I. scapularis* densities have been found in grasslands and other open canopy environments, where humidity conditions may be unsuitable for their survival (Ginsberg et al., 2020, Mathisson et al., 2021).

Greater host abundances may variably influence tick populations locally. In regions with long‐established tick populations, higher densities of mammal hosts, especially white‐tailed deer (*Odocoileus virginianus*), are expected to increase tick abundances due to additional contact opportunities (Dobson, 2014; Estrada‐Peña & De La Fuente, 2014; Levi et al., 2016). In addition, the relative abundance of white‐footed mice (*Peromyscus leucopus*) may also impact the immature *I. scapularis* abundance feeding on small mammals (Bouchard et al., 2011, 2013; Werden et al., 2014). In contrast, tick–host interactions in areas with emergent tick populations, such as those near the poleward range edge, may be dynamic and lead to variable *I. scapularis* abundances because of limited host availability (Dobson, 2014; Millien et al., 2023). Yet, these relationships may be unclear if only tick dragging were conducted (Dobson, 2014). Therefore, the complementary use of small mammal trapping with tick dragging during active surveillance may provide a better metric to assess the relationships between *I. scapularis* and small mammal communities.

The diversity of mammal species may also affect tick abundances due to the quality of blood meal hosts present within the community. Different mammal species may vary in their ability to successfully feed ticks (LoGiudice et al., 2003; Mather et al., 1989). Small mammal hosts, such as white‐footed mice, chipmunks (*Tamias striatus*), and shrews (*Blarina brevicauda* and *Sorex cinereus*), play an important role in tick survival by successfully feeding a greater abundance of immature ticks (LoGiudice et al., 2003, Mather et al., 1989). Certain mid‐size and large mammals, such as raccoons (*Procyon lotor*) and white‐tailed deer, may also impact tick abundance by feeding large burdens of ticks, and act as key reproductive hosts for adult *I. scapularis* (LoGiudice et al., 2003). However, *I. scapularis* abundance may also be restricted by host‐specific behaviors, where lower quality hosts may kill ticks while grooming or exhibit physiological immune responses that result in ticks unsuccessfully feeding (Jones et al., 2015; Keesing et al., 2009; Levin & Fish, 1998).

Recently, an Earth observation‐informed framework was designed that combines high‐resolution environment data with traditional vector surveillance data for climate‐related risk assessments and mapping of disease vectors, such as *I. scapularis*, at varying geographic scales (Kotchi et al., 2019, 2021). These high‐resolution environmental factors may be derived from satellite‐based remote sensing imagery and direct‐contact sensors from meteorological towers (Kotchi et al., 2019, 2021). However, this framework does not incorporate information related to hosts, which are important predictors of the abundance and distribution of *I. scapularis*. Here, we incorporated high‐resolution environmental and host surveillance data to provide a better understanding of the dynamics between the abiotic and biotic factors impacting questing *I. scapularis* abundance. More specifically, we evaluated the concurrent impact of abiotic factors derived from remote‐sensing imagery and meteorological towers in addition to biotic factors obtained through host active surveillance in the field on questing *I. scapularis* abundance along the northward edge of their range in Ontario and Quebec, Canada. We predicted that the questing *I. scapularis* abundance may be variably influenced by combinations of abiotic and biotic factors. We expected increased *I. scapularis* abundances at localities with more suitable environmental habitats, including those with warmer temperatures, greater precipitation or lower evapotranspiration, greater snow accumulation, and greater vegetation greenness. We also expected variable relationships between questing *I. scapularis* with small mammal abundance, the relative abundance of *P. leucopus*, and mammal species richness, which may relate to the degree of establishment of tick and host populations locally. Our study is the first to combine high‐resolution environmental and host‐related factors to determine the mechanisms driving the local *I. scapularis* abundance, as a means to better anticipate the spread of tick populations and tick‐borne pathogens in Canada.

## 2 MATERIALS AND METHODS

### 2.1 Field sampling

**FIGURE 1:**
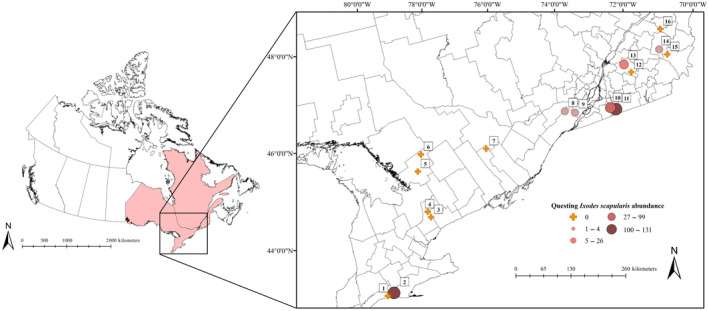
Questing *Ixodes scapularis* abundance across our study sites in Central Canada. Circle size and color represent the questing abundance of *I. scapularis*, with larger circles in darker colors denoting greater tick abundance. Sites with no *I. scapularis* are represented by orange crosses. (1) 3 Ridges Farm, (2) New New Age Farm, (3) North Tract, (4) Brown Hill Tract, (5) Upjohn Nature Reserve, (6) Dyer Memorial Nature Reserve, (7) Rose Hill Nature Reserve, (8) Kirkview Farm, (9) Saint‐Polycarpe, (10), Saint‐Valentin, (11) Henryville, (12) Lefebvre, (13) Parc du Sanctuaire Saint‐Majorique, (14) Serpentine‐de‐Coleraine Ecological Reserve, (15) Frontenac National Park, and (16) Saint‐Sylvestre.

Sixteen forested sites were visited for sampling in July and August 2019 in Ontario and Quebec, Canada (Figure 1). These sites were selected based on distinct levels of estimated Lyme disease risk related to the abundances and life stages of *I. scapularis* present locally as defined by the Institut national de santé publique du Québec (2018) and Public Health Ontario (2018), which ranged from possible to significant risk (Table S2). At each site, three grids of 40 m by 70 m were delineated in contiguous forest areas suitable for tick dragging, small mammal trapping, and the placement of trail cameras. These grids were maximally separated by 100 meters due to geographic barriers (e.g., streams or park trails).

Within each grid, a 1 m^2^ cotton flannel was dragged over the low‐lying vegetation along four 70‐meter‐long transects to collect questing ticks. Every 10 m, flannels were checked, and ticks were removed and placed into microvials with 95% ethanol. Larvae were pooled, while nymphs and adults were kept individually. Tick specimens were identified to the species with dichotomous keys (Egizi et al., 2019; Lindquist et al., 2016).

At each site, a total of 84 Sherman live traps (H.B. Sherman Traps, Inc., Florida, USA) were placed along four parallel transects within each grid for three consecutive nights. Targeted small mammal species included mice (*Peromyscus leucopus* and *P. maniculatus*), shrews (*Blarina brevicauda* and *Sorex cinereus*), voles (*Microtus pennsylvanicus* and *Myodes gapperi*), and jumping mice (*Napaeozapus insignis* and *Zapus hudsonius*). In the afternoon, we placed bait (peanut butter and oatmeal), a water source (apple piece), and nesting material (cotton ball) in each trap, which were checked the following morning. Non‐targeted species and juveniles were immediately released on site. Individuals of targeted species were euthanized by isoflurane inhalation followed by cervical dislocation. One red squirrel (*Tamiasciurus hudsonicus*) and two hairy‐tailed moles (*Parascalops breweri*) were also euthanized due to severe injuries. Small mammals were screened for feeding ticks, and liver tissues were dissected from collected specimens and placed into microvials with 95% ethanol. Using molecular methods based on a modified protocol of Tessier et al. (2004), each *Peromyscus* specimen was identified to the species using their liver tissues (Supplementary Methods). All samples were accessioned at the Redpath Museum, McGill University (Table S3). Ethical approval and permits were issued by McGill University (AUP No. 2019‐8086), the Ministère des Forêts, de la Faune et des Parcs (SEG permit No. 2019‐06‐04‐008‐00‐S‐F), and the Ministry of Natural Resources and Forestry (WSCA No. 1093495).

Nine trail cameras (Force‐10, SpyPoint Inc., Quebec, Canada) were concurrently placed on trees 1 m above the ground inside our sampling area and set to take three consecutive photos without delay for each detection. We identified each mammal host species seen in the photographs taken by the camera traps. Birds, domestic pets, humans, and unidentified individuals were not included in the dataset.

At each site, questing *I. scapularis* abundance was calculated as the sum of questing ticks collected along transects while tick dragging. The total number of collected small mammals was used as a proxy for the abundance of small mammals locally. The relative abundance of *P. leucopus* was estimated as the number of collected *P. leucopus* individuals at each site divided by the local abundance of collected small mammals. The number of mammal host species was quantified as the number of the distinct species found via small mammal trapping and detected in camera photographs.

### 2.2 Meteorological data

We extracted historical data from December 2018 to December 2019 for precipitation (PRECIP) in mm and snow on the ground (SNOW) in cm using nearby local meteorological towers (Environment and Climate Change Canada, 2021). The distance from our sites to nearby meteorological towers ranged from 7.13 to 36.21 km (Table S4). We removed estimated and flagged values from the dataset. We calculated monthly mean PRECIP (July or August) dependent on when field surveys were conducted at each locality. We determined the accumulated SNOW by calculating the difference in snow on the ground between 2 days (i.e., (*n* + 1) − n) from December 2018 to June 2019. If the difference was greater than 0, then this value was used; otherwise, the value was set to 0. These calculated values were then summed from the start of winter to the end of spring.

### 2.3 Remote sensing data

Broad‐scale remote sensing data were used to extract historical values for temperature, evapotranspiration, and vegetation greenness across our sites. All GIS analyses were conducted in ArcMap version 10.7.1. (Esri Inc., 2019). Shapefiles and rasters were re‐projected into the NAD 83 Canada Atlas Lambert projection.

We extracted three version 6 data products for two adjacent tiles (12,4 and 13,4) from December 2018 to December 2019 using the Moderate Resolution Imaging Spectroradiometer (MODIS) on board NASA's Terra satellite. MOD11A2 is an average 8‐day land surface temperature (LST) at a 1‐km spatial resolution (Wan et al., 2015). MOD13A3 provided a monthly average of the enhanced vegetation index (EVI) at a 1‐km spatial resolution (Didan, 2015). MOD16A2 is an 8‐day composite of total evapotranspiration (TE) at a 500‐m spatial resolution (Running et al., 2017).

Raster processing of MODIS data included format conversion, re‐projection, clipping, resampling, mosaicking, applying scale factors, converting measurements (if applicable), creating masks based on pixel quality control, and calculating zonal statistics for each of our sites. HDF‐EOS files were converted to TIFF files using NASA's HEG Conversion Tool (2019). Rasters were resampled with cubic convolution to 500 m by 500 m cells using the *Resample* tool. The two tiles were then mosaicked together by Julian date using the *Mosaic to New Raster* tool. Using the *Raster Calculator* tool, scale factors and measurement conversions were applied. For LST, day and night values were converted to °C from K by multiplying the values by a scale factor of 0.02 and subtracting 273.15. Values were multiplied by a 0.0001 scale factor for EVI and a 0.1 scale factor for TE.

Quality assessment layers were decoded using the *MODIS Decode Quality* tool from the ArcGIS MODIS Python toolbox. Valid pixels were extracted using the *Extract by Attributes* tool. For LST, valid pixels included a low LST error (<= 2 K), a low emissivity error (<= 0.02), and good or other quality data (Wan et al., 2015). For EVI, valid pixels included a VI usefulness of the two highest quality categories (0000 and 0001) and pixel reliability with good or marginal data (Didan, 2015). For TE, valid pixels included those of good quality using the main algorithm, detectors fine for up to 50% of channels, and a cloud state that was clear or not defined, but appeared to be clear (Running et al., 2017). Finally, we clipped valid pixels to a 1‐km buffer around each site in Central Canada using the *Clip* tool.

Using the *Extract by Attributes* tool, masks of the valid pixels were applied to remove outlier values. Day and night LST values less than −50°C and greater than 100°C and TE values below 0 or greater than 3276.1 (fill values) were discarded. We then calculated zonal statistics (mean, maximum, and minimum) of LST, EVI, and TE from December 2018 to December 2019 within the 1‐km buffer using the *Zonal Statistics as Table* tool. The daily, weekly, and monthly mean LST as well as the winter minimum LST and summer maximum LST were calculated for all our sites. Summer mean TE and summer mean EVI were calculated from June 2019 to September 2019.

### 2.4 Statistical analyses

All statistical analyses were performed in R version 4.1.1 (R Core Team, 2021). Using the *rcorr* function in the *Hmisc* package (Harrell Jr., 2021), Spearman correlations were conducted to assess if correlation coefficients were above 0.50 and if significant collinearity (*p* < .05) was present between abiotic factors (monthly mean PRECIP, accumulated SNOW, monthly mean LST, minimum winter LST, maximum summer LST, summer mean TE, and summer mean EVI) and biotic factors (small mammal abundance, relative abundance of *P. leucopus*, and mammal species richness). This type of correlation was selected due to its non‐parametric nature, which could assess potential non‐linear relationships between abiotic and biotic factors. Due to high collinearity, minimum winter LST and maximum summer LST were dropped from further analyses. Similarly, correlated biotic variables were to be run separately in further analyses to not violate statistical assumptions. Due to data limitations, summer mean TE and summer mean EVI were removed as independent variables, as values could only be calculated for 13 of our 16 sites. The remaining abiotic and biotic factors were each centered and standardized with the *scale* function.

Spatial autocorrelation among abiotic and biotic variables was assessed with Moran's I with an inverse distance weights matrix using the *moran. test* function in the *spdep* package (Bivand & Wong, 2018).

Finally, we conducted count regression generalized additive models (GAM) using the *gam* function in the *mgcv* package with a negative binomial family (Wood, 2017) to investigate the concurrent impact of abiotic and biotic factors on questing *I. scapularis* abundance. GAMs were chosen for our analyses as we expected that the biotic factors may exhibit variable relationships with *I. scapularis* abundance. A negative binomial family rather than a Poisson family was selected based on AIC, rootograms, and Pearson dispersion parameters. All GAMs were fitted using penalized thin plate spline regressions (bs = “tp”) and a double penalty approach to account for sparse data and be used for variable selection (Marra & Wood, 2011). We used a REML method for our GAMs, as this method is more robust to under‐smoothing and small sample sizes (Wood, 2017). Using the argument select = TRUE in the *gam* function, a double penalty approach can penalize function components in both the range and null space, which can then be shrunk to zero (Marra & Wood, 2011). As a result, this approach allows for model selection without requiring a stepwise selection procedure and uses fewer effective degrees of freedom (Marra & Wood, 2011). A smoothed interaction of latitude and longitude was used in all models to account for spatial autocorrelation (Marra & Wood, 2011). All models were inspected for goodness of fit using the *gam. check* function, AIC, adjusted *R* ^2^, and deviance explained. We also assessed if collinearity was present between the model's smooth terms using the *concurvity* function (Wood, 2017). If concurvity was high, we then ran simplified models that only included the significant terms to determine if any predictors should be removed.

Three GAMs were conducted to determine how abiotic and biotic factors were concurrently affecting questing *I. scapularis* abundance across our sites. Model 1 assessed the impact of the small mammal community on questing *I. scapularis* abundance by including small mammal abundance, monthly mean LST, monthly mean PRECIP, accumulated SNOW, and spatial autocorrelation as independent variables. A high‐leverage outlier (Site 9: Saint‐Polycarpe) was detected after running this model. As a result, we removed this outlier and ran a subsequent Model 1 using the remainder of the data. The formula for Model 1 is as follows: Questing *I. scapularis* abundance ~ s(Small mammal abundance) + s(Monthly mean LST) + s(Monthly mean PRECIP) + s(Accumulated SNOW) + s(Longitude, Latitude). Model 2 assessed the relative contribution of *P. leucopus* within the small mammal community by including the relative abundance of *P. leucopus*, monthly mean LST, monthly mean PRECIP, accumulated SNOW, and spatial autocorrelation as independent variables. The formula for Model 2 is as follows: Questing *I. scapularis* abundance ~ s(Relative abundance of *P. leucopus*) + s(Monthly mean LST) + s(Monthly mean PRECIP) + s(Accumulated SNOW) + s(Longitude, Latitude). Model 3 assessed the mammal community as a whole using mammal species richness, monthly mean LST, monthly mean PRECIP, and spatial autocorrelation as independent variables. Accumulated SNOW was not used in Model 3 due to high collinearity with mammal species richness. The formula for Model 3 is as follows: Questing *I. scapularis* abundance ~ s(Mammal species richness) + s(Monthly mean LST) + s(Monthly mean PRECIP) + s(Longitude, Latitude).

## 3 RESULTS

### 3.1 Field sampled *I. scapularis* and mammal hosts

**FIGURE 2:**
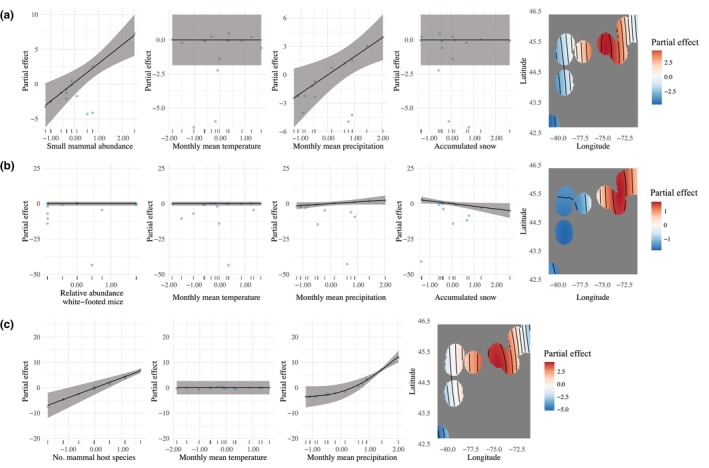
Partial effects of generalized additive models with questing *Ixodes scapularis* abundance as a response variable with a smoothed interaction of latitude and longitude to account for spatial autocorrelation. Panels (a), (b), and (c) correspond to the graphical representations of Model 1 without the high‐leverage outlier, Model 2, and Model 3, respectively. Smoothed abiotic factors include monthly mean land surface temperature (LST), monthly mean precipitation (PRECIP), and accumulated snow on the ground (SNOW). Smoothed biotic factors include small mammal abundance, relative abundance of *P. leucopus*, and mammal species richness. Shaded areas represent the 95% credible intervals of the model and points represent the residual values. (a) Although significant relationships for questing *I. scapularis* abundance were found for small mammal abundance and monthly mean PRECIP in Model 1, this model does not have predictive power based on graphical visualizations and the adjusted *R*‐squared value. *R*‐sq (adj.) = −31.300, deviance explained = 82.20%. (b) Positive and negative relationships were found between questing *I. scapularis* abundance with monthly mean PRECIP and accumulated SNOW, respectively, but no effect was found for the relative abundance of *P. leucopus*. *R*‐sq (adj.) = .445, deviance explained = 57.60%. (c) Mammal species richness and monthly mean precipitation both significantly impacted questing *I. scapularis* abundance. *R*‐sq (adj.) = .994, deviance explained = 99.70%.

We collected 382 questing *I. scapularis* from eight of our 16 sites ranging from 2 to 164 ticks (Figure 1; Table S5). These questing *I. scapularis* included 255 larvae (29 pools), 126 nymphs, and one adult male. We found that questing *I. scapularis* abundance increased with decreasing longitude, likely due to the majority of *I. scapularis* being collected during field surveys in eastern Ontario and southern Quebec at sites 8 to 11 (Figure 2).

We collected a total of 105 individuals with live traps, which belonged to twelve small mammal species (Table S5). *Peromyscus leucopus* was the most abundant species (31.4% of collected individuals; *n* = 33) and was present at nine of our 16 sites. Other small mammals collected, in decreasing order of abundance, included *N. insignis* (22.8%; *n* = 24), *B. brevicauda* (16.2%; *n* = 17), *M. gapperi* (15.2%; *n* = 16), *P. maniculatus* (10.5%; *n* = 11), *P. breweri* (1.9%; *n* = 2), *S. cinereus* (1.0%; *n* = 1), and *M. pennsylvanicus* (1.0%; *n* = 1). Small mammal abundance ranged from 1 to 18 individuals, and the relative abundance of *P. leucopus* at each site varied from 0.00 to 1.00 (Table S5). Five mammal host species were identified from camera photographs, which included squirrels (*Sciurus carolinensis*), chipmunks (*T. striatus*), white‐tailed deer (*O. virginianus*), raccoons (*P. lotor*), and coyotes (*Canis latrans*). Between 2 and 8 mammal species were detected locally via small mammal trapping and in camera photographs (Table S5).

### 3.2 Collinearity of abiotic and biotic factors

Several factors were found to be significantly correlated with each other (Figure S1). Mammal species richness was significantly correlated with small mammal abundance (*r* = .54, *p* < .05), accumulated SNOW (*r* = −.56, *p* < .05), and minimum winter LST (*r* = .59, *p* < .05). Maximum summer LST was significantly correlated with the relative abundance of *P. leucopus* (*r* = .65, *p* < .01) and accumulated SNOW (*r* = −.50, *p* < .05). Finally, summer mean TE and summer mean EVI were significantly correlated (*r* = −.71, *p* < .05). As a result of significant collinearity or data limitations, minimum winter LST, maximum summer LST, summer mean TE, and summer mean EVI were removed from further analyses. To limit multicollinearity, separate analyses were conducted for each biotic factor with the remaining abiotic factors.

### 3.3 Spatial autocorrelation

We detected spatial autocorrelation for several variables, which included questing *I. scapularis* abundance (Moran's *I* = 0.206, *p* < .05), monthly mean PRECIP (Moran's *I* = 0.389, *p* < .01), accumulated SNOW (Moran's *I* = 0.226, *p* < .05), and monthly mean LST (Moran's *I* = 0.385, *p* < .01). However, we did not detect any spatial autocorrelation for the biotic factors: small mammal abundance (Moran's *I* = −0.199, *p* = .797), relative abundance of *P. leucopus* (Moran's *I* = 0.072, *p* = .207), and mammal species richness (Moran's *I* = 0.138, *p* = .115).

### 3.4 Effect of abiotic and biotic factors on questing *I. scapularis* abundance

We found that the questing *I. scapularis* abundance was modulated in different ways depending on the abiotic and biotic factors that were assessed. A high‐leverage outlier (Site 9: Saint‐Polycarpe) was detected in Model 1, which incited our removal of this outlier to re‐assess Model 1 with the remainder of the data. In Model 1, small mammal abundance and monthly mean PRECIP had a significant effect on questing *I. scapularis* abundance (Figure 2a; Table S6). However, even with the removal of this outlier, we obtained a negative adjusted *R*‐squared value, indicating that this model did not have predictive power (Table S6). In Model 2, we found a linear positive relationship for monthly mean PRECIP and a linear negative relationship for accumulated SNOW with questing *I. scapularis* abundance, respectively (Figure 2b; Table S7). However, we did not find a relationship between the relative abundance of *P. leucopus* and questing *I. scapularis* abundance in this model (Figure 2b; Table S7). In Model 3, both mammal species richness and monthly mean PRECIP were found to have positive effects on questing *I. scapularis* abundance (Figure 2c; Table S8). The concurvity of all our models were assessed through simplified models using only significant factors and the spatial autocorrelation term. These simplified models indicated that we did not need to remove any predictor variables when fitting our data.

## 4 DISCUSSION

Using a combination of high‐resolution environmental data and field‐based sampling, we provide evidence that combinations of abiotic and biotic factors drive questing *I. scapularis* abundance across our sites in Central Canada. We first found that greater precipitation and less accumulated snow were associated with increased questing *I. scapularis* abundance, but no effect was found for the relative abundance of white‐footed mice. We also found that questing *I. scapularis* abundance was most positively influenced by monthly mean precipitation and mammal species richness, where tick abundances increased with greater precipitation and greater numbers of mammal species locally. These results highlight the importance of incorporating host active surveillance data with high‐resolution environmental data when assessing which abiotic and biotic factors are impacting questing *I. scapularis* abundance.

### 4.1 Influence of biotic factors on *I. scapularis* abundance

We found that larger abundances of questing *I. scapularis* were associated with greater mammal species richness locally (Figure 2c). Areas with more diverse mammal communities may have increased *I. scapularis* abundances, but only if host abundances increase with species richness allowing for greater tick–host contact rates (Luis et al., 2018; Ogden & Tsao, 2009). Here, a significant positive correlation between small mammal abundance and mammal species richness was found, which may provide additional contact and feeding opportunities for *I. scapularis*. This relationship may be especially impacted by mid‐size and large mammals, such as raccoons and white‐tailed deer, that can successfully feed large burdens of ticks including immature and adult *I. scapularis* (LoGiudice et al., 2003; Werden et al., 2014). These mammal hosts may also be important for the local dispersal and establishment of *I. scapularis* to locations further north. Moreover, the addition or loss of mammal host species locally due to predation or interspecific competition may have variable impacts on tick abundances, especially for *I. scapularis* populations that have not yet established at their northward range edge (Levi et al., 2016). In addition, *I. scapularis* may be variably affected if lower quality mammal hosts are present that may kill or unsuccessfully feed ticks due to host‐specific behaviors including grooming or physiological immune responses (Jones et al., 2015; Keesing et al., 2009; Levin & Fish, 1998).

In contrast, we did not find relationships between questing *I. scapularis* abundance with small mammal abundance nor the relative abundance of *P. leucopus* in Central Canada. Areas with long‐established populations of *I. scapularis* were associated with higher abundances of small mammal hosts. In southern Quebec, increased abundances of infected *I. scapularis* have also been associated with increased abundances of small mammals across the same geographic extent as our study (Millien et al., 2023). However, it may be that avian hosts or larger mammals, such as white‐tailed deer, play a larger role in the maintenance of tick populations across our sites, where increasing densities of these hosts may lead to increased abundances of questing *I. scapularis* locally (Bouchard et al., 2011, 2013; Brisson et al., 2008; LoGiudice et al., 2003; Mather et al., 1989). In addition, fluctuating host densities within the small mammal community may result in variable *I. scapularis* abundances due to limited tick‐host contacts (Dobson, 2014; Linske et al., 2018; Luis et al., 2018). This variability in tick–host interactions may be especially discernible in areas where *I. scapularis* may not have fully established, such as those populations located near the northward range edge. For example, several of our sites in northeastern Ontario and southeastern Quebec did not harbor any *P. leucopus* or *I. scapularis*, which likely impacted this relationship. It may be that populations of *P. leucopus* and *I. scapularis* are not yet present in these areas or remain scarce, but they are expected to become established in the near future with their northward geographic range expansion (Clow, Leighton, et al., 2017; Ripoche et al., 2022; Roy‐Dufresne et al., 2013; Simon et al., 2014).

### 4.2 Impacts of abiotic factors on tick and host populations

Greater amounts of precipitation were associated with increased abundances of questing *I. scapularis* (Figure 2b,c). Sufficient levels of moisture and precipitation may sustain suitable humidity levels within the microclimate for the survival of *I. scapularis* (Berger, Ginsberg, Dugas, et al., 2014; Berger, Ginsberg, Gonzalez, & Mather, 2014; Dumas et al., 2022). At localities with greater amounts of precipitation, *I. scapularis* may not be required to mitigate their desiccation through behavioral changes (Vail & Smith, 2002). As a result, these tick vectors are more likely to be actively questing within their environment for a suitable host rather than remaining close to the leaf litter with limited activity (Burtis et al., 2016; Vail & Smith, 2002). In addition, greater amounts of precipitation may also lead to increased vegetation greenness, yet the spatial heterogeneity of precipitation may variably impact this relationship locally (Jiang et al., 2016). Here, we found a weak negative relationship between monthly mean PRECIP and summer mean EVI (*r* = −.38), which may be primarily due to several sites in Ontario with no *I. scapularis* present that had low to moderate levels of vegetation greenness. Therefore, we may find a positive association between precipitation, vegetation, and questing *I. scapularis* abundance in the future as tick populations become established at these localities.

Surprisingly, we found that increased levels of accumulated snow were associated with decreased abundances of questing *I. scapularis* (Figure 2b). Snow cover alone or in combination with leaf litter typically leads to increased overwintering survival, resulting in increased abundances of questing *I. scapularis* in the subsequent summer (Hayes et al., 2015; Linske et al., 2019; Volk et al., 2022). However, the localities with the highest questing *I. scapularis* abundances were at our southernmost sites in Ontario and Quebec, which were associated with the lowest amounts of accumulated snow. Although these areas may have experienced milder winters, it does not seem that an increased mortality risk due to inoculative freezing impacted subsequent summer *I. scapularis* abundances (Eisen et al., 2016; Linske et al., 2019; Volk et al., 2022). In contrast, localities at the northern range edge experienced large snow accumulations, yet *I. scapularis* populations were not present or were present in very low abundances the following summer. Therefore, it is possible that an additional metric other than accumulated snow may be beneficial to completely capture the microclimatic winter conditions.

Changes in climatic conditions, such as precipitation, snow, or vegetation, may also impact the movements and poleward range expansions of mammal hosts, altering the abundance and distribution of *I. scapularis* (Diuk‐Wasser et al., 2021; Ogden & Lindsay, 2016). Small mammal hosts may disperse short distances searching for food resources, such as acorns or seed crops, in nearby forested areas, which may result in fluctuations of local tick populations (Borgmann‐Winter et al., 2021; Marrotte et al., 2017; Sullivan et al., 2023). The summer following high abundances of acorns or other seed crops have been associated with higher abundances and greater overwintering survival in *Peromyscus* mice, resulting in a lagged increase in *I. scapularis* abundance (Falls et al., 2007; Ostfeld et al., 2018; Sullivan et al., 2023). In addition, decreased winter severity may lead to greater movements and habitat use of mammal hosts, especially for white‐tailed deer (Dawe & Boutin, 2016; Fisher et al., 2020). With climate warming, milder winters have been associated with greater poleward range expansions in white‐tailed deer and white‐footed mice, which have assisted in the range expansion of *I. scapularis* to new poleward locations (Dawe & Boutin, 2016; Fisher et al., 2020; Kennedy‐Slaney et al., 2018; Roy‐Dufresne et al., 2013; Simon et al., 2014).

Finally, unexplored factors, such as microclimatic conditions or avian host communities, may be affecting questing *I. scapularis* abundances across our sites in Central Canada. Increasing the number of sampled sites through larger scale surveillance efforts may provide additional insights on the impacts of abiotic and biotic factors on questing *I. scapularis* across a broader spatial region. The abundance of *I. scapularis* as well as abiotic and biotic factors may change through time, which will require further study to explore the interannual dynamics of this system. As a result, future surveillance efforts should be conducted across a broader region incorporating a greater number of sites over several consecutive years to increase the spatial and temporal sampling periods. In addition, future analyses should use raster‐based products at finer temporal and spatial resolutions (e.g., daily metrics at a local scale) to simultaneously assess the relative impact of high‐resolution abiotic and biotic factors on questing *I. scapularis*.

### 4.3 Implications for surveillance efforts

Our study demonstrates that host active surveillance data should be incorporated with high‐resolution abiotic variables to comprehensively assess the relationships and dynamics between questing *I. scapularis* and host populations. The current design of the Earth observation‐informed framework (Kotchi et al., 2019) excludes vertebrate hosts, which are a key player in tick–host–pathogen disease systems, especially at the poleward range edge. We propose the incorporation of host active surveillance data in this framework or future studies that rely on high‐resolution environmental data, as it may provide greater knowledge of the driving mechanisms of increased *I. scapularis* abundances across large geographic areas or time frames at varying scales (Kotchi et al., 2019). Public health agencies may then be better informed as to which areas may have increased abundances of *I. scapularis* and hosts and should therefore be targeted by active surveillance or control efforts (Kotchi et al., 2019). We encourage future studies to use a combination of biotic factors obtained during field‐based surveys, such as the abundance and diversity of mammal hosts, in addition to high‐resolution abiotic factors derived from remote‐sensing imagery and meteorological tower data to better assess the spread of tick populations and tick‐borne pathogens in Canada.

## 5 CONCLUSION

We incorporated host active surveillance data obtained through field‐based sampling with high‐resolution, multitemporal environmental data derived from remote‐sensing imagery and meteorological towers to evaluate the concurrent effects of abiotic and biotic factors on questing *Ixodes scapularis* abundance. Combinations of abiotic and biotic factors were identified as important drivers of abundances of questing *I. scapularis*. Positive and negative linear relationships were found for questing *I. scapularis* abundance with monthly mean precipitation and accumulated snow, respectively, but no effect was found for the relative abundance of white‐footed mice. Positive relationships were found between questing *I. scapularis* abundance with monthly mean precipitation and mammal species richness, where increased tick abundances occurred with greater precipitation and greater mammal species richness locally. Therefore, future studies assessing *I. scapularis* should incorporate host surveillance data to enable more sensitive tests of the relative importance of the abiotic environmental conditions on the abundance and diversity of hosts and disease vectors, such as *I. scapularis*. Such relationships may prove especially important in areas with emerging populations of medically important tick vectors. Disentangling the influence of abiotic and biotic factors remains critical to understanding how the environment and host community affect tick populations and tick‐borne disease risk in Canada and elsewhere.

## AUTHOR CONTRIBUTIONS

**Kirsten E. Crandall:** Conceptualization (equal); data curation (lead); formal analysis (lead); methodology (lead); visualization (lead); writing – original draft (lead); writing – review and editing (equal). **Virginie Millien:** Conceptualization (equal); funding acquisition (equal); supervision (equal); writing – review and editing (equal). **Jeremy T. Kerr:** Conceptualization (equal); funding acquisition (equal); supervision (equal); writing – review and editing (equal).

## CONFLICT OF INTEREST STATEMENT

The authors declare no conflict of interest.

## Supporting information


Data S1

## Data Availability

Data supporting the findings of this study are summarized in the Appendix. The Python and R codes required to extract data and conduct analyses in addition to the raw field date and extracted environmental data from remote sensing satellites and meteorological towers are publicly available on Dryad (https://doi.org/10.5061/dryad.cc2fqz6c8).
