# Supplementary material for: High‐resolution environmental and host‐related factors impacting questing Ixodes scapularis at their northern range edge
Source: Ecol Evol. 2024 Feb 21;14(2):e10855. doi: 10.1002/ece3.10855 (PMC10879908; doi:10.1002/ece3.10855)
Supplement: Supplementary file 1 — Data S1 [file ECE3-14-e10855-s001.docx]

**Appendix**

**Table S1. **The expected directional relationships of abiotic and biotic factors on *Ixodes scapularis* abundance in Ontario and Quebec, Canada.

| **Factor** | **Expected**  **directional change** | **Reasoning** | **References** |
| --- | --- | --- | --- |
| Temperature | ↑ Tick abundance | With climate warming, higher temperatures are expected to increase the interstadial development rates and the length of seasonal activity periods, resulting in increased tick abundances. | Ogden et al. 2004, Eisen et al. 2016, Ogden and Lindsay 2016 |
|  | ↓ Tick abundance | Limited interstadial development and reduced activity is expected when temperatures within the microclimate are outside the optimal thermal thresholds of tick populations (e.g., too low or too high). With extreme hot and cold temperatures, increased mortality rates may occur due to water loss or inoculative freezing, respectively. |  |
| Precipitation | ↑ Tick abundance | The presence and activity of ticks may increase at moderate levels of precipitation due to sufficient humidity levels. | Eisen et al. 2016, Ogden and Lindsay 2016, Burtis et al. 2016 |
|  | ↓ Tick abundance | Low precipitation may lead to desiccation stress in ticks, thereby limiting their questing activity and survival. |  |
| Snow cover | ↑ Tick abundance | Greater snow cover alone or in combination with leaf litter may increase the overwintering survival of tick populations. | Hayes et al. 2015, Eisen et al. 2016, Linske et al. 2019, Volk et al. 2022 |
|  | ↓ Tick abundance | Milder winters may lead to reduced snow cover, which may expose ticks to inoculative freezing, even in the presence of leaf litter, resulting in decreased survival. |  |
| Vegetation | ↑ Tick abundance | Areas with dense vegetation may be more suitable habitats for tick populations, leading to greater tick densities. | Schulze and Jordan 2001, Clow et al. 2017, Ginsberg et al. 2020, Mathisson et al. 2021 |
|  | ↓ Tick abundance | Areas with less dense vegetation may lead to decreased survival for tick populations due to low relative humidity conditions. |  |
| Mammal host abundance | Variable tick abundance | Greater densities of mammal hosts, especially in areas with long-established tick populations, may lead to more contact opportunities between hosts and ticks, thus reducing the time for finding a suitable host and limiting mortality. | Dobson 2014, Estrada-Peña and De La Fuente 2014 |
|  |  | Areas with tick populations that have dynamic tick-host interactions may have variable tick abundances. These areas may have limited abundances of key mammal hosts, such as white-footed mice or white-tailed deer, thus affecting tick survival and development. |  |
| Mammal host diversity | Variable tick abundance | The composition of mammal communities may affect the ability of ticks to successfully feed and further develop because of their quality as blood meal hosts. Adding host species to the local community may increase the number of feeding opportunities for ticks, resulting in increased tick abundance. In addition, the presence of certain mammal hosts, such as white-footed mice and white-tailed deer, may increase tick abundances.  However, host-specific differences, such as physiological immune responses, movements, and grooming behaviours, may impact tick burdens and lead to variable tick abundances locally. | Mather et al. 1989, LoGiudice et al. 2003, Jones et al. 2015 |

**References**

Burtis, J. C., P. Sullivan, T. Levi, K. Oggenfuss, T. J. Fahey, and R. S. Ostfeld. 2016. The impact of temperature and precipitation on blacklegged tick activity and Lyme disease incidence in endemic and emerging regions. Parasites & Vectors 9:606.

Clow, K., N. H. Ogden, L. R. Lindsay, P. Michel, D. L. Pearl, and C. M. Jardine. 2017. The influence of abiotic and biotic factors on the invasion of *Ixodes scapularis* in Ontario, Canada. Ticks and Tick-borne Diseases 8:554–563.

Dobson, A. D. M. 2014. History and complexity in tick-host dynamics: Discrepancies between ‘real’ and ‘visible’ tick populations. Parasites & Vectors 7:231.

Eisen, R., L. Eisen, N. Ogden, and C. Beard. 2016. Linkages of weather and climate with *Ixodes scapularis* and *Ixodes pacificus* (Acari: Ixodidae), enzootic transmission of *Borrelia burgdorferi*, and Lyme disease in North America. Journal of Medical Entomology 53:250–261.

Estrada-Peña, A., and J. De La Fuente. 2014. The ecology of ticks and epidemiology of tick-borne viral diseases. Antiviral Research 108:104–128.

Ginsberg, H. S., E. L. Rulison, J. L. Miller, G. Pang, I. M. Arsnoe, G. J. Hickling, N. H. Ogden, R. A. LeBrun, and J. I. Tsao. 2020. Local abundance of *Ixodes scapularis* in forests: Effects of environmental moisture, vegetation characteristics, and host abundance. Ticks and Tick-borne Diseases 11:101271.

Hayes, L. E., J. A. Scott, and K. C. Stafford. 2015. Influences of weather on *Ixodes scapularis* nymphal densities at long-term study sites in Connecticut. Ticks and Tick-borne Diseases 6:258–266.

Jones, C. R., J. L. Brunner, G. A. Scoles, and J. P. Owen. 2015. Factors affecting larval tick feeding success: Host, density and time. Parasites & Vectors 8:340.

Linske, M. A., K. C. Stafford, S. C. Williams, C. B. Lubelczyk, M. Welch, and E. F. Henderson. 2019. Impacts of deciduous leaf litter and snow presence on nymphal *Ixodes scapularis* (Acari: Ixodidae) overwintering survival in coastal New England, USA. Insects 10:227.

LoGiudice, K., R. S. Ostfeld, K. A. Schmidt, and F. Keesing. 2003. The ecology of infectious disease: Effects of host diversity and community composition on Lyme disease risk. Proceedings of the National Academy of Sciences of the United States of America 100:567–571.

Mather, T. N., M. L. Wilson, S. I. Moore, J. M. C. Ribeiro, and A. Spielman. 1989. Comparing the relative potential of rodents as reservoirs of the Lyme disease spirochete (*Borrelia burgdorferi*). American Journal of Epidemiology 130:143–150.

Mathisson, D. C., S. M. Kross, M. I. Palmer, and M. A. Diuk-Wasser. 2021. Effect of vegetation on the abundance of tick vectors in the northeastern United States: A review of the literature. Journal of Medical Entomology 58:2030–2037.

Ogden, N., L. Lindsay, G. Beauchamp, D. Charron, A. Maarouf, C. O’Callaghan, D. Waltner-Toews, and I. Barker. 2004. Investigation of relationships between temperature and developmental rates of tick *Ixodes scapularis* (Acari: Ixodidae) in the laboratory and field. Journal of Medical Entomology 41:622–633.

Ogden, N. H., and L. R. Lindsay. 2016. Effects of climate and climate change on vectors and vector-borne diseases: Ticks are different. Trends in Parasitology 32:646–656.

Schulze, T. L., and R. A. Jordan. 2001. Effects of habitat structure on the retention of *Ixodes scapularis* and *Amblyomma mericanum* (Acari: Ixodidae) adults during drag sampling surveys. Journal of Medical Entomology 38:606–608.

Volk, M. R., C. B. Lubelczyk, J. C. Johnston, D. L. Levesque, and A. M. Gardner. 2022. Microclimate conditions alter *Ixodes scapularis* (Acari: Ixodidae) overwinter survival across climate gradients in Maine, United States. Ticks and Tick-borne Diseases 13:101872.

**Supplementary Methods**

DNA extractions and polymerase chain reactions (PCR) were conducted by Geneticks Inc. to identify the species of *Peromyscus* specimens. Mammalian liver tissues were extracted using the Thermo Scientific GeneJET Genomic Purification Kit (Thermo Fisher Scientific, Massachusetts, United States). Modifications to the Mammalian Tissue and Rodent Tail Genomic DNA Purification protocol (Protocol A, 2016) were made, which included using 10 mg of liver, removing residual solution with extra centrifugation step 9, and not adding more elution buffer after sitting for 5 minutes prior to centrifugation. Nested PCRs were run using species-specific COIII primers with an initial denaturation time of 5 minutes (Tessier, Noël, and Lapointe 2004). PCR products were then run on 3% agarose gel, stained with Eco-Stain (Bio Basic, Markham, Canada), and visualized using a blue light transilluminator.

**Reference**

Tessier, N., S. Noël, and F.-J. Lapointe. 2004. A new method to discriminate the deer mouse (*Peromyscus maniculatus*) from the white-footed mouse (*Peromyscus leucopus*) using species-specific primers in multiplex PCR. Canadian Journal of Zoology 82:1832–1835.

**Table S2.** Estimated site-level Lyme disease risk based on the 2018 Lyme disease risk maps in Ontario and Quebec as well as the local abundances and life stages of questing and feeding *Ixodes scapularis*. In Ontario, estimated risk areas are calculated as a 20 km radius from the centre location of questing *I. scapularis* found through tick dragging (Public Health Ontario 2018). In Quebec, municipality risk levels were associated with human Lyme disease cases as well as the abundances and life stages of *I. scapularis* ticks detected through passive and active surveillance (Institut national de santé publique du Québec 2018). Due to significant differences in the provincial classifications of Lyme disease risk areas, we provide two variables related to estimated local Lyme disease risk: (1) a binary variable (0 = possible risk, 1 = present risk) based on definitions by Public Health Ontario and (2) a 3-category variable (1 = possible risk, 2 = present risk, 3 = significant risk) based on definitions by Institut national de santé publique du Québec.

| Site ID | Site | Estimated Lyme disease risk  (possible or present) | Estimated Lyme disease risk  (possible, present, or significant) |
| --- | --- | --- | --- |
| 1 | 3 Ridges Farm | 0 | 1 |
| 2 | New New Age Farm | 1 | 3 |
| 3 | North Tract | 1 | 2 |
| 4 | Brown Hill Tract | 1 | 2 |
| 5 | Upjohn Nature Reserve | 0 | 1 |
| 6 | Dyer Memorial Nature Reserve | 0 | 1 |
| 7 | Rose Hill Nature Reserve | 0 | 1 |
| 8 | Kirkview Farm | 1 | 2 |
| 9 | Saint-Polycarpe | 1 | 2 |
| 10 | Saint-Valentin | 1 | 3 |
| 11 | Henryville | 1 | 3 |
| 12 | Lefebvre | 1 | 2 |
| 13 | Parc du Sanctuaire Saint-Majorique | 1 | 3 |
| 14 | Serpentine-de-Coleraine Ecological Reserve | 1 | 2 |
| 15 | Frontenac National Park | 1 | 2 |
| 16 | Saint-Sylvestre | 1 | 2 |

| Questing *I. scapularis* abundance | Feeding *I. scapularis* abundance | Life stage(s) present |
| --- | --- | --- |
| 0 | 0 | None |
| 131 | 33 | Larvae and nymphs |
| 0 | 2 | Nymphs |
| 0 | 0 | None |
| 0 | 0 | None |
| 0 | 0 | None |
| 0 | 0 | None |
| 4 | 1 | Nymphs |
| 1 | 1 | Larvae and nymphs |
| 99 | 18 | Larvae and nymphs |
| 118 | 7 | Larvae, nymphs, and adults |
| 0 | 0 | None |
| 26 | 3 | Larvae and nymphs |
| 3 | 0 | Larvae and nymphs |
| 0 | 0 | None |
| 0 | 0 | None |

**References**

Institut national de santé publique du Québec. 2018. Carte de risque d’acquisition de la maladie de Lyme selon les municipalités du Québec, 2018. Gouvernement du Québec.

Public Health Ontario. 2018. Ontario Lyme disease map 2018: Estimated risk areas. Queen’s Printer for Ontario.

**Table S3. **List of small mammal specimens collected during our field surveys and accessioned at the Redpath Museum, McGill University (Montreal, Quebec, Canada).

| Accession number | Species | Site ID | Location | Latitude (ºN) | Longitude (ºW) |
| --- | --- | --- | --- | --- | --- |
| RMMA20210812 | *Parascalops breweri* | 1 | 3 Ridges Ecological Farm | 42.697 | -81.026 |
| RMMA20210824 | *Peromyscus leucopus* | 1 | 3 Ridges Ecological Farm | 42.697 | -81.026 |
| RMMA20210816 | *Peromyscus leucopus* | 2 | New New Age Farm | 42.73 | -80.835 |
| RMMA20210825 | *Napaeozapus insignis* | 2 | New New Age Farm | 42.73 | -80.835 |
| RMMA20210813 | *Napaeozapus insignis* | 2 | New New Age Farm | 42.731 | -80.837 |
| RMMA20210814 | *Peromyscus leucopus* | 2 | New New Age Farm | 42.731 | -80.836 |
| RMMA20210817 | *Napaeozapus insignis* | 2 | New New Age Farm | 42.731 | -80.837 |
| RMMA20210818 | *Peromyscus leucopus* | 2 | New New Age Farm | 42.731 | -80.836 |
| RMMA20210819 | *Napaeozapus insignis* | 2 | New New Age Farm | 42.731 | -80.837 |
| RMMA20210820 | *Peromyscus leucopus* | 2 | New New Age Farm | 42.731 | -80.836 |
| RMMA20210821 | *Peromyscus leucopus* | 2 | New New Age Farm | 42.731 | -80.836 |
| RMMA20210822 | *Napaeozapus insignis* | 2 | New New Age Farm | 42.731 | -80.836 |
| RMMA20210823 | *Napaeozapus insignis* | 2 | New New Age Farm | 42.731 | -80.837 |
| RMMA20210826 | *Parascalops breweri* | 2 | New New Age Farm | 42.731 | -80.835 |
| RMMA20210827 | *Napaeozapus insignis* | 2 | New New Age Farm | 42.731 | -80.837 |
| RMMA20210828 | *Napaeozapus insignis* | 2 | New New Age Farm | 42.731 | -80.837 |
| RMMA20210829 | *Napaeozapus insignis* | 2 | New New Age Farm | 42.731 | -80.837 |
| RMMA20210830 | *Napaeozapus insignis* | 2 | New New Age Farm | 42.731 | -80.837 |
| RMMA20210831 | *Napaeozapus insignis* | 2 | New New Age Farm | 42.731 | -80.837 |
| RMMA20210815 | *Peromyscus leucopus* | 2 | New New Age Farm | 42.771 | -80.835 |
| RMMA20210838 | *Napaeozapus insignis* | 3 | North Tract | 44.081 | -79.311 |
| RMMA20210840 | *Microtus pennsylvanicus* | 3 | North Tract | 44.081 | -79.31 |
| RMMA20210841 | *Napaeozapus insignis* | 3 | North Tract | 44.081 | -79.311 |
| RMMA20210839 | *Napaeozapus insignis* | 3 | North Tract | 44.09 | -79.311 |
| RMMA20210833 | *Napaeozapus insignis* | 3 | North Tract | 44.081 | -79.31 |
| RMMA20210832 | *Peromyscus leucopus* | 3 | North Tract | 44.082 | -79.313 |
| RMMA20210834 | *Peromyscus leucopus* | 4 | Brown Hill Tract | 44.209 | -79.366 |
| RMMA20210835 | *Peromyscus leucopus* | 4 | Brown Hill Tract | 44.209 | -79.366 |
| RMMA20210842 | *Peromyscus leucopus* | 4 | Brown Hill Tract | 44.209 | -79.366 |
| RMMA20210836 | *Peromyscus leucopus* | 4 | Brown Hill Tract | 44.21 | -79.366 |
| RMMA20210837 | *Peromyscus leucopus* | 4 | Brown Hill Tract | 44.21 | -79.366 |
| RMMA20210809 | *Peromyscus leucopus* | 5 | Upjohn Nature Reserve | 45.076 | -79.36 |
| RMMA20210810 | *Peromyscus leucopus* | 5 | Upjohn Nature Reserve | 45.076 | -79.36 |
| RMMA20210808 | *Peromyscus maniculatus* | 6 | Dyer Memorial Nature Reserve | 45.404 | -79.149 |
| RMMA20210811 | *Napaeozapus insignis* | 6 | Dyer Memorial Nature Reserve | 45.404 | -79.149 |
| RMMA20210803 | *Myodes gapperi* | 7 | Rose Hill Nature Reserve | 45.159 | -77.226 |
| RMMA20210806 | *Myodes gapperi* | 7 | Rose Hill Nature Reserve | 45.159 | -77.227 |
| RMMA20210807 | *Peromyscus maniculatus* | 7 | Rose Hill Nature Reserve | 45.159 | -77.227 |
| RMMA20210801 | *Peromyscus maniculatus* | 7 | Rose Hill Nature Reserve | 45.16 | -77.227 |
| RMMA20210802 | *Napaeozapus insignis* | 7 | Rose Hill Nature Reserve | 45.16 | -77.226 |
| RMMA20210804 | *Peromyscus maniculatus* | 7 | Rose Hill Nature Reserve | 45.16 | -77.227 |
| RMMA20210805 | *Napaeozapus insignis* | 7 | Rose Hill Nature Reserve | 45.16 | -77.226 |
| RMMA20210843 | *Peromyscus leucopus* | 8 | Kirkview Farm | 45.422 | -74.67 |
| RMMA20210844 | *Peromyscus leucopus* | 8 | Kirkview Farm | 45.422 | -74.67 |
| RMMA20210846 | *Peromyscus leucopus* | 8 | Kirkview Farm | 45.422 | -74.67 |
| RMMA20210847 | *Peromyscus leucopus* | 8 | Kirkview Farm | 45.422 | -74.67 |
| RMMA20210848 | *Peromyscus leucopus* | 8 | Kirkview Farm | 45.422 | -74.67 |
| RMMA20210852 | *Blarina brevicauda* | 9 | Saint-Polycarpe | 45.329 | -74.394 |
| RMMA20210855 | *Peromyscus leucopus* | 9 | Saint-Polycarpe | 45.329 | -74.394 |
| RMMA20210856 | *Peromyscus leucopus* | 9 | Saint-Polycarpe | 45.329 | -74.394 |
| RMMA20210845 | *Peromyscus leucopus* | 9 | Saint-Polycarpe | 45.33 | -74.394 |
| RMMA20210849 | *Peromyscus leucopus* | 9 | Saint-Polycarpe | 45.33 | -74.394 |
| RMMA20210850 | *Peromyscus leucopus* | 9 | Saint-Polycarpe | 45.33 | -74.394 |
| RMMA20210851 | *Peromyscus leucopus* | 9 | Saint-Polycarpe | 45.33 | -74.394 |
| RMMA20210853 | *Peromyscus leucopus* | 9 | Saint-Polycarpe | 45.33 | -74.394 |
| RMMA20210854 | *Blarina brevicauda* | 9 | Saint-Polycarpe | 45.33 | -74.394 |
| RMMA20210857 | *Peromyscus leucopus* | 9 | Saint-Polycarpe | 45.33 | -74.394 |
| RMMA20210858 | *Blarina brevicauda* | 9 | Saint-Polycarpe | 45.33 | -74.394 |
| RMMA20210859 | *Blarina brevicauda* | 9 | Saint-Polycarpe | 45.33 | -74.394 |
| RMMA20210860 | *Blarina brevicauda* | 9 | Saint-Polycarpe | 45.33 | -74.394 |
| RMMA20210866 | *Peromyscus leucopus* | 10 | Saint-Valentin | 45.185 | -73.348 |
| RMMA20210867 | *Peromyscus leucopus* | 10 | Saint-Valentin | 45.185 | -73.348 |
| RMMA20210868 | *Peromyscus leucopus* | 10 | Saint-Valentin | 45.185 | -73.347 |
| RMMA20210869 | *Myodes gapperi* | 10 | Saint-Valentin | 45.185 | -73.347 |
| RMMA20210870 | *Napaeozapus insignis* | 10 | Saint-Valentin | 45.185 | -73.347 |
| RMMA20210871 | *Myodes gapperi* | 10 | Saint-Valentin | 45.185 | -73.347 |
| RMMA20210872 | *Myodes gapperi* | 10 | Saint-Valentin | 45.185 | -73.347 |
| RMMA20210874 | *Myodes gapperi* | 10 | Saint-Valentin | 45.185 | -73.348 |
| RMMA20210875 | *Myodes gapperi* | 10 | Saint-Valentin | 45.185 | -73.347 |
| RMMA20210861 | *Peromyscus maniculatus* | 11 | Henryville | 45.117 | -73.212 |
| RMMA20210862 | *Peromyscus maniculatus* | 11 | Henryville | 45.117 | -73.212 |
| RMMA20210864 | *Peromyscus maniculatus* | 11 | Henryville | 45.117 | -73.21 |
| RMMA20210873 | *Myodes gapperi* | 11 | Henryville | 45.117 | -73.211 |
| RMMA20210863 | *Peromyscus leucopus* | 11 | Henryville | 45.118 | -73.211 |
| RMMA20210865 | *Peromyscus leucopus* | 11 | Henryville | 45.118 | -73.211 |
| RMMA20210883 | *Myodes gapperi* | 12 | Lefebvre | 45.738 | -72.406 |
| RMMA20210884 | *Myodes gapperi* | 12 | Lefebvre | 45.738 | -72.406 |
| RMMA20210885 | *Blarina brevicauda* | 12 | Lefebvre | 45.738 | -72.406 |
| RMMA20210886 | *Blarina brevicauda* | 12 | Lefebvre | 45.738 | -72.406 |
| RMMA20210892 | *Blarina brevicauda* | 12 | Lefebvre | 45.738 | -72.406 |
| RMMA20210888 | *Myodes gapperi* | 13 | Parc du Sanctuaire Saint-Majorique | 45.943 | 72.53 |
| RMMA20210891 | *Napaeozapus insignis* | 13 | Parc du Sanctuaire Saint-Majorique | 45.943 | -72.529 |
| RMMA20210893 | *Blarina brevicauda* | 13 | Parc du Sanctuaire Saint-Majorique | 45.943 | -72.53 |
| RMMA20210894 | *Myodes gapperi* | 13 | Parc du Sanctuaire Saint-Majorique | 45.943 | -72.531 |
| RMMA20210881 | *Peromyscus maniculatus* | 13 | Parc du Sanctuaire Saint-Majorique | 45.944 | -72.53 |
| RMMA20210882 | *Myodes gapperi* | 13 | Parc du Sanctuaire Saint-Majorique | 45.944 | -72.531 |
| RMMA20210887 | *Napaeozapus insignis* | 13 | Parc du Sanctuaire Saint-Majorique | 45.944 | -72.53 |
| RMMA20210889 | *Napaeozapus insignis* | 13 | Parc du Sanctuaire Saint-Majorique | 45.944 | -72.53 |
| RMMA20210890 | *Myodes gapperi* | 13 | Parc du Sanctuaire Saint-Majorique | 45.944 | -72.529 |
| RMMA20210895 | *Napaeozapus insignis* | 13 | Parc du Sanctuaire Saint-Majorique | 45.944 | -72.53 |
| RMMA20210877 | *Peromyscus maniculatus* | 14 | Serpentine-de-Coleraine Ecological Reserve | 45.978 | -71.37 |
| RMMA20210878 | *Peromyscus maniculatus* | 14 | Serpentine-de-Coleraine Ecological Reserve | 45.978 | -71.371 |
| RMMA20210879 | *Myodes gapperi* | 14 | Serpentine-de-Coleraine Ecological Reserve | 45.978 | -71.37 |
| RMMA20210880 | *Peromyscus maniculatus* | 14 | Serpentine-de-Coleraine Ecological Reserve | 45.978 | -71.37 |
| RMMA20210876 | *Sorex cinereus* | 15 | Frontenac National Park | 45.815 | -71.203 |
| RMMA20210896 | *Blarina brevicauda* | 16 | Saint-Sylvestre | 46.368 | -71.119 |
| RMMA20210897 | *Blarina brevicauda* | 16 | Saint-Sylvestre | 46.368 | -71.118 |
| RMMA20210898 | *Blarina brevicauda* | 16 | Saint-Sylvestre | 46.368 | -71.118 |
| RMMA20210899 | *Napaeozapus insignis* | 16 | Saint-Sylvestre | 46.368 | -71.118 |
| RMMA202108100 | *Blarina brevicauda* | 16 | Saint-Sylvestre | 46.368 | -71.118 |
| RMMA202108101 | *Blarina brevicauda* | 16 | Saint-Sylvestre | 46.368 | -71.119 |
| RMMA202108102 | *Myodes gapperi* | 16 | Saint-Sylvestre | 46.368 | -71.119 |
| RMMA202108103 | *Blarina brevicauda* | 16 | Saint-Sylvestre | 46.368 | -71.118 |
| RMMA202108104 | *Blarina brevicauda* | 16 | Saint-Sylvestre | 46.368 | -71.118 |
| RMMA202108105 | *Blarina brevicauda* | 16 | Saint-Sylvestre | 46.368 | -71.118 |

**Table S4.** Summarized local abiotic factors across our sites in Central Canada. High-resolution environmental factors included monthly mean precipitation (PRECIP), accumulated snow on the ground (SNOW), monthly mean land surface temperature (LST), winter minimum LST, summer maximum LST, summer mean total evapotranspiration (TE), and summer mean enhanced vegetation index (EVI), which were derived from Environment and Climate Change Canada weather towers and NASA’s MODIS Terra remote sensing satellites. Latitudinal and longitudinal coordinates in degrees were used to account for spatial autocorrelation.

| Site ID | Site | Latitude (ºN) | Longitude (ºW) | Weather tower ID | Distance of  tower to site (km) |
| --- | --- | --- | --- | --- | --- |
| 1 | 3 Ridges Farm | 42.70 | -81.03 | 6137362 | 16.19 |
| 2 | New New Age Farm | 42.73 | -80.84 | 6138270 | 16.95 |
| 3 | North Tract | 44.08 | -79.31 | 6110480 | 20.80 |
| 4 | Brown Hill Tract | 44.21 | -79.36 | 6110480 | 7.13 |
| 5 | Upjohn Nature Reserve | 45.08 | -79.36 | 6110607 | 7.50 |
| 6 | Dyer Memorial Nature Reserve | 45.40 | -79.15 | 6117981 | 26.49 |
| 7 | Rose Hill Nature Reserve | 45.16 | -77.22 | 6105762 | 36.21 |
| 8 | Kirkview Farm | 45.42 | -74.67 | 7016470 | 25.27 |
| 9 | Saint-Polycarpe | 45.33 | -74.39 | 7011947 | 17.52 |
| 10 | Saint-Valentin | 45.18 | -73.35 | 7026916 | 11.06 |
| 11 | Henryville | 45.12 | -73.21 | 7026734 | 10.78 |
| 12 | Lefebvre | 45.74 | -72.41 | 7027470 | 13.67 |
| 13 | Parc du Sanctuaire Saint-Majorique | 45.94 | -72.53 | 7027470 | 12.76 |
| 14 | Serpentine-de-Coleraine Ecological Reserve | 45.98 | -71.37 | 7028442 | 11.11 |
| 15 | Frontenac National Park | 45.82 | -71.20 | 7024320 | 24.46 |
| 16 | Saint-Sylvestre | 46.37 | -71.12 | 7027656 | 15.27 |

| Monthly mean PRECIP (mm) | Accumulated SNOW (cm) | Monthly mean LST (°C) | Winter minimum LST (°C) | Summer maximum LST (°C) |
| --- | --- | --- | --- | --- |
| 3.28 | 43.00 | 20.53 | -26.35 | 28.53 |
| 4.39 | 45.00 | 24.63 | -14.75 | 25.05 |
| 0.97 | 104.00 | 16.20 | -21.21 | 26.21 |
| 0.97 | 104.00 | 16.07 | -44.05 | 26.15 |
| 0.77 | 141.00 | 20.16 | -29.91 | 23.99 |
| 1.70 | 299.00 | 22.90 | -25.55 | 23.73 |
| 1.22 | 94.00 | 18.50 | -22.61 | 22.99 |
| 1.33 | 104.00 | 26.41 | -24.03 | 26.41 |
| 2.17 | 107.00 | 25.10 | -30.39 | 34.83 |
| 3.66 | 124.00 | 18.10 | -32.05 | 25.65 |
| 2.64 | 98.00 | 20.55 | -24.49 | 29.95 |
| 1.82 | 135.00 | 18.84 | -25.15 | 25.11 |
| 1.82 | 135.00 | 17.40 | -20.71 | 21.87 |
| 5.19 | 216.00 | 10.30 | -25.79 | 24.53 |
| 3.67 | 175.00 | 12.02 | -26.83 | 23.37 |
| 3.47 | 181.00 | 14.18 | -24.17 | 24.79 |

| Summer mean TE (mm) | Summer mean EVI |
| --- | --- |
| 599.94 | 0.59 |
| 528.69 | 0.60 |
| 522.19 | 0.47 |
| 93.93 | 0.53 |
| 49.49 | 0.64 |
| 312.29 | 0.59 |
| 1572.77 | 0.20 |
| 1791.37 | 0.19 |
| NA | 0.06 |
| NA | 0.15 |
| 1194.53 | 0.14 |
| 1319.40 | NA |
| 2215.20 | 0.16 |
| 936.20 | 0.10 |
| 1490.59 | NA |
| NA | NA |

**Table S5.** Summarized local biotic factors found via small mammal trapping and trail cameras across our sites in Central Canada. Biotic factors included small mammal abundance, the relative abundance of white-footed mice (*Peromycus leucopus*), and the number of mammal host species (or mammal species richness). Questing *I. scapularis* abundance was calculated as the sum of questing ticks collected along transects at each site while tick dragging. The total number of collected small mammals was used as a proxy for the abundance of small mammals locally. The relative abundance of *P. leucopus* was estimated as the number of collected *P. leucopus* individuals at each site divided by the local abundance of collected small mammals. Mammal species richness was quantified as the number of the distinct species found via small mammal trapping and detected in camera photographs. Latitudinal and longitudinal coordinates in degrees were used to account for spatial autocorrelation.

| Site ID | Site | Latitude (ºN) | Longitude (ºW) | Questing *I. scapularis* abundance |
| --- | --- | --- | --- | --- |
| 1 | 3 Ridges Farm | 42.70 | -81.03 | 0 |
| 2 | New New Age Farm | 42.73 | -80.84 | 131 |
| 3 | North Tract | 44.08 | -79.31 | 0 |
| 4 | Brown Hill Tract | 44.21 | -79.36 | 0 |
| 5 | Upjohn Nature Reserve | 45.08 | -79.36 | 0 |
| 6 | Dyer Memorial Nature Reserve | 45.4 | -79.15 | 0 |
| 7 | Rose Hill Nature Reserve | 45.16 | -77.22 | 0 |
| 8 | Kirkview Farm | 45.42 | -74.67 | 4 |
| 9 | Saint-Polycarpe | 45.33 | -74.39 | 1 |
| 10 | Saint-Valentin | 45.18 | -73.35 | 99 |
| 11 | Henryville | 45.12 | -73.21 | 118 |
| 12 | Lefebvre | 45.74 | -72.41 | 0 |
| 13 | Parc du Sanctuaire Saint-Majorique | 45.94 | -72.53 | 26 |
| 14 | Serpentine-de-Coleraine Ecological Reserve | 45.98 | -71.37 | 3 |
| 15 | Frontenac National Park | 45.82 | -71.20 | 0 |
| 16 | Saint-Sylvestre | 46.37 | -71.12 | 0 |

| Small mammal abundance | Relative abundance  *P. leucopus* | No. host species  (small mammal trapping) | No. host species  (camera) | No. host species (trapping and camera) |
| --- | --- | --- | --- | --- |
| 2 | 0.500 | 4 | 4 | 7 |
| 18 | 0.333 | 4 | 3 | 7 |
| 6 | 0.167 | 4 | 1 | 5 |
| 5 | 1.000 | 1 | 2 | 3 |
| 2 | 1.000 | 2 | 1 | 3 |
| 2 | 0.000 | 2 | 1 | 3 |
| 7 | 0.000 | 4 | 1 | 5 |
| 5 | 1.000 | 2 | 4 | 6 |
| 13 | 0.615 | 3 | 2 | 5 |
| 9 | 0.333 | 4 | 1 | 5 |
| 6 | 0.333 | 5 | 2 | 7 |
| 5 | 0.000 | 3 | 2 | 5 |
| 10 | 0.000 | 7 | 1 | 8 |
| 4 | 0.000 | 2 | 0 | 2 |
| 1 | 0.000 | 3 | 1 | 4 |
| 10 | 0.000 | 5 | 1 | 6 |

**
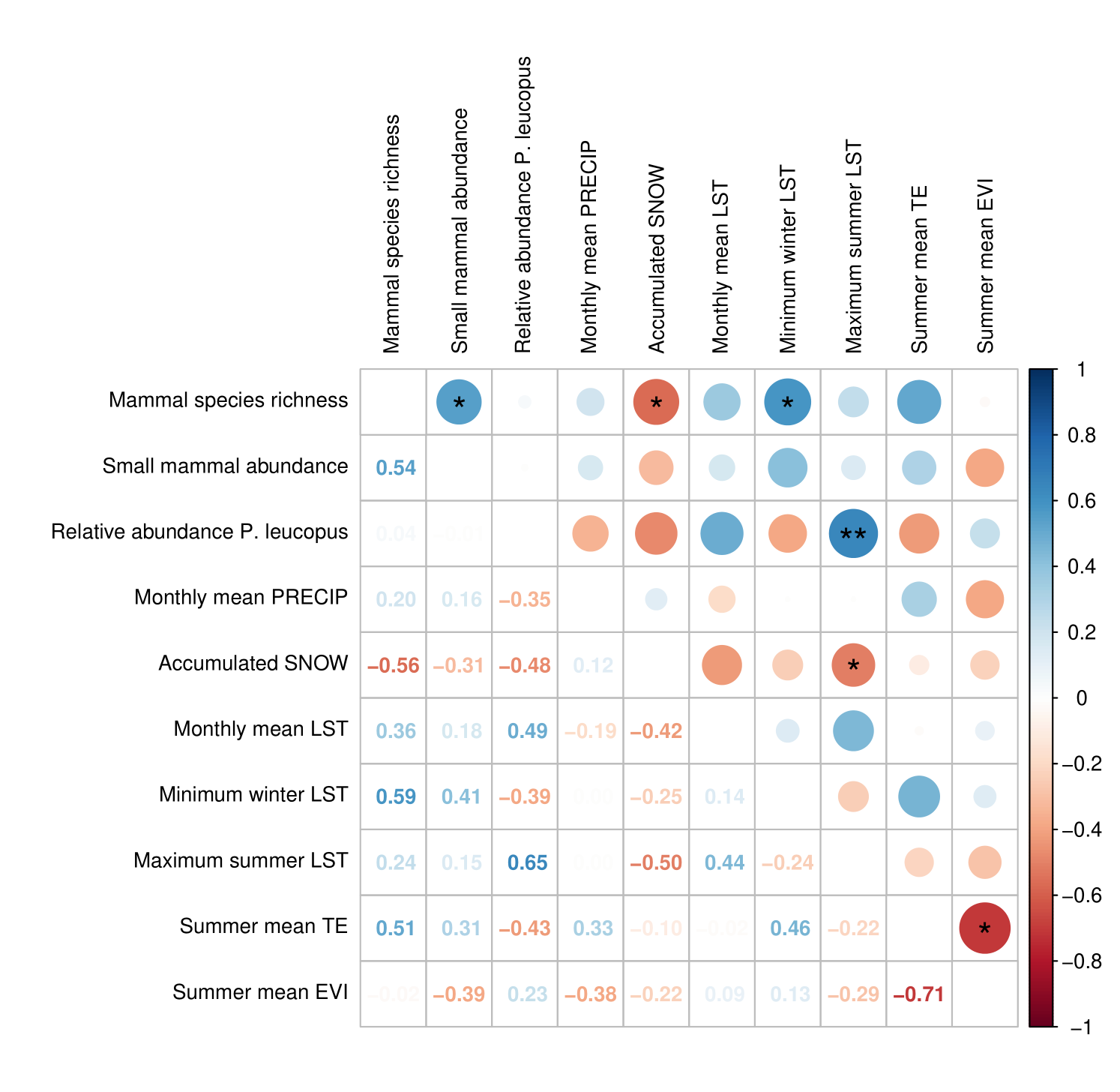
**

**Figure S1.** Correlogram showing Spearman correlations among abiotic and biotic factors across our sites in Ontario and Quebec, Canada. Spearman correlation coefficients are represented with the scale and below the diagonal (< 0 = negatively correlated, > 0 = positively correlated). Red circles and coefficients are related to negative correlations, while blue circles and coefficients represent positive correlations. Circles increase in size with larger coefficient values. Significance of terms is denoted by *** P < 0.001, ** P < 0.01, * P < 0.05.

**Table S6.** Parameter estimates and fit measures for Model 1 assessing the effect of smoothed abiotic factors and small mammal abundance as well as the smooth interaction of spatial coordinates on the questing *Ixodes scapularis* abundance in Central Canada. The model formula is Questing *I. scapularis* abundance ~ s(Small mammal abundance) + s(Monthly mean LST) + s(Monthly mean PRECIP) + s(Accumulated SNOW) + s(Longitude, Latitude). Significance of terms is denoted by *** P < 0.001, ** P < 0.01, * P < 0.05. LST: land surface temperature; PRECIP: precipitation; SNOW: snow on the ground. Model 1 fit measures include an AIC of 75.374, 82.20% deviance explained, an adjusted R^2^ of -31.300, and a REML of 37.857.

| Parametric term | Coefficient | Std. Error | *z* value | *P*-value |
| --- | --- | --- | --- | --- |
| Intercept | -0.803 | 0.949 | -0.846 | 0.397 |

| Smooth terms | Estimated values | Std. Errors | Confidence intervals | Est. df | Ref. df. | Chi. Sq | *P*-value |
| --- | --- | --- | --- | --- | --- | --- | --- |
| s(Small mammal abundance) | -1.224 - 2.516 | 0.727 – 1.619 | -6.214 - 9.991 | 0.942 | 2 | 13.184 | < 0.001*** |
| s(Monthly mean LST) | -1.876 - 1.652 | 0.949 | -1.860 - 1.860 | 0.000 | 2 | 0.000 | 0.849 |
| s(Monthly mean PRECIP) | -1.236 - 2.031 | 0.813 - 1.583 | -5.551 - 6.353 | 0.921 | 2 | 7.928 | 0.003** |
| s(Accumulated SNOW) | -1.395 - 2.638 | 0.949 | -1.860 - 1.860 | 0.000 | 2 | 0.000 | 0.365 |
| s(Longitude, Latitude) | -81.03° to -71.12°  42.70° to 46.37° | 0.620 - 2.091 | -8.876 - 7.004 | 0.946 | 3 | 13.981 | < 0.001*** |

**Table S7.** Parameter estimates and fit measures for Model 2 assessing the effect of smoothed abiotic factors and the relative abundance of *Peromyscus leucopus* as well as the smooth interaction of spatial coordinates on the questing *Ixodes scapularis* abundance in Central Canada. The model formula is Questing *I. scapularis* abundance ~ s(Relative abundance *P. leucopus*) + s(Monthly mean LST) + s(Monthly mean PRECIP) + s(Accumulated SNOW) + s(Longitude, Latitude). Significance of terms is denoted by *** P < 0.001, ** P < 0.01, * P < 0.05. LST: land surface temperature; PRECIP: precipitation; SNOW: snow on the ground. Model 2 fit measures include an AIC of 90.065, 57.60% deviance explained, an adjusted R^2^ of 0.445, and a REML of 42.811.

| Parametric term | Coefficient | Std. Error | *z* value | *P*-value |
| --- | --- | --- | --- | --- |
| Intercept | 0.930 | 0.708 | 1.312 | 0.189 |

| Smooth terms | Estimated values | Std. Errors | Confidence intervals | Est. df | Ref. df. | Chi. Sq | *P*-value |
| --- | --- | --- | --- | --- | --- | --- | --- |
| s(Relative abundance *P. leucopus*) | -0.853 - 1.731 | 0.708 | -1.388 - 1.388 | 0.000 | 3.000 | 0.000 | 0.644 |
| s(Monthly mean LST) | -1.876 - 1.652 | 0.708 | -1.388 - 1.388 | 0.000 | 2.000 | 0.000 | 0.469 |
| s(Monthly mean PRECIP) | -1.236 - 2.031 | 0.702 - 1.620 | -3.967 - 5.679 | 0.788 | 2.000 | 2.612 | 0.043* |
| s(Accumulated SNOW) | -1.395 - 2.638 | 0.669 - 2.631 | -10.085 - 4.948 | 0.794 | 2.000 | 4.549 | 0.013* |
| s(Longitude, Latitude) | -81.03° to -71.12°  42.70° to 46.37° | 0.861 - 1.586 | -4.723 – 3.454 | 1.452 | 5.000 | 4.665 | 0.024* |

**Table S8.** Parameter estimates and fit measures for Model 3 assessing the effect of smoothed abiotic factors and mammal species richness as well as the smooth interaction of spatial coordinates on the questing *Ixodes scapularis* abundance in Central Canada. Accumulated snow was excluded in this model due to high collinearity with mammal species richness. The model formula is Questing *I. scapularis* abundance ~ s(No. mammal species) + s(Monthly mean LST) + s(Monthly mean PRECIP) + s(Longitude, Latitude). Significance of terms is denoted by *** P < 0.001, ** P < 0.01, * P < 0.05. PRECIP: precipitation; LST: land surface temperature. Model 3 fit measures include an AIC of 53.597, 99.70% deviance explained, an adjusted R^2^ of 0.994, and a REML of 37.258.

| Parametric term | Coefficient | Std. Error | *z* value | *P*-value |
| --- | --- | --- | --- | --- |
| Intercept | -1.813 | 1.344 | -1.349 | 0.177 |

| Smooth terms | Estimated values | Std. Errors | Confidence intervals | Est. df | Ref. df. | Chi. Sq | *P*-value |
| --- | --- | --- | --- | --- | --- | --- | --- |
| s(No. mammal species) | -1.769 - 1.697 | 0.432 - 2.529 | -11.903 - 7.51 | 0.975 | 3.000 | 32.583 | 0.000*** |
| s(Monthly mean LST) | -1.876 - 1.652 | 1.344 | -2.634 - 2.634 | 0.000 | 2.000 | 0.000 | 0.493 |
| s(Monthly mean PRECIP) | -1.236 - 2.031 | 0.330 - 2.130 | -7.768 - 14.624 | 1.912 | 2.000 | 32.666 | 0.000*** |
| s(Longitude, Latitude) | -81.03° to -71.12°  42.70° to 46.37° | 0.259 - 3.203 | -9.054 - 5.842 | 2.363 | 5.000 | 45.582 | 0.000*** |
